# Comparison of Icare Pro Tonometry and Icare One Tonometry Measurements in Healthy Eyes

**DOI:** 10.4274/tjo.galenos.2018.06787

**Published:** 2019-06-27

**Authors:** Hüseyin Mayalı, Çağlar Sarıgül, Emin Kurt, Özcan Rasim Kayıkçıoğlu, Süleyman Sami İlker

**Affiliations:** 1 Manisa Celal Bayar University Faculty of Medicine, Department of Ophthalmology, Manisa, Turkey

**Keywords:** Glaucoma, intraocular pressure, Icare, tonometry

## Abstract

**Objectives:**

To compare intraocular pressure (IOP) measurements obtained with the Icare Pro tonometer used in clinical practice, and the Icare One self-tonometer.

**Materials and Methods:**

Fifty-two eyes of 52 healthy, right-handed individuals with no prior intraocular surgery or ocular trauma, structural ocular pathology, or systemic disease were evaluated. IOP was first measured using the Icare Pro tonometer. The participants were then told how to use the Icare One tonometer and asked to measure their own IOP. The results were analyzed statistically using SPSS v.24.

**Results:**

Of the 52 healthy participants, 16 (30.7%) were male and 36 (69.3%) were female. Their mean age was 31.6±6.3 (23-47) years. Mean IOP measured with the Icare Pro was 17.10±6.2 (11.5-25.2) mmHg, and the mean self-measured IOP with Icare One was 14.01±3.4 (7-24) mmHg. When the two methods were compared using Levene’s t test, there was a significant mean difference of -3.08±0.6 (95% confidence interval: -4.39 -1.78; p<0.001).

**Conclusion:**

In this study, there was a significant difference between the IOP measurements we made using the Icare Pro and the participants’ self-measured IOP using the Icare One, with the latter being relatively lower. This may be related to the fact that the participants were unfamiliar with using the Icare One. Although the Icare One is a promising tool for glaucoma patients to self-monitor their IOP, further studies are needed.

## Introduction

The accurate measurement and regular monitoring of intraocular pressure (IOP) are critical in the diagnosis, follow-up, and treatment of glaucoma. Various devices are currently used to measure IOP, but the Goldmann applanation tonometer (GAT) is still the gold standard.^1,2^

The Icare Pro is a small, portable, easy-to-use tonometer that operates on the principle of rebound measurement and does not require topical anesthesia. Measurements are obtained by striking the central cornea with a single-use probe on the device’s tip. The average of six measurements obtained by the device is displayed as the IOP value.^2^ The Icare One tonometer was designed to allow individuals to measure their own IOP.^3^

The development of home tonometers patients can use to assess their IOP is important for evaluating the effectiveness of antiglaucoma therapy in reducing IOP.^4^

This study was conducted to compare measurements obtained with the Icare Pro tonometer and Icare One tonometers in healthy eyes.

## Materials and Methods

The study was carried out in accordance with the principles of clinical research set forth in the Declaration of Helsinki and was approved by the ethics committee of Manisa Celal Bayar University Faculty of Medicine. The study included 52 right eyes of 52 healthy, right-handed individuals with no history of intraocular surgery or trauma and no structural ocular pathology or systemic disease. All participants underwent best corrected visual acuity assessment, slit-lamp anterior segment examination, and fundus examination.

IOP measurements were obtained with the Icare Pro tonometer, changing probes between each measurement.

Measurements were performed an average of 6 times from the central cornea with the device held approximately 4-8 mm from the right eye without topical anesthesia, and the average IOP value was determined.

### Statistical Analyses

Ten minutes after measuring with the Icare Pro tonometer, the participants were told how to use the Icare One tonometer and were instructed to measure the IOP of their right eye using their right hand. The results were statistically analyzed using SPSS 24. The two methods were compared using Levene’s test with level of significance accepted as p<0.05.

## Results

Of the 52 healthy individuals included in the study, 16 (30.7%) were male and 36 (69.3%) were female. Their mean age was 31.6±6.3 (23-47) years. Mean IOP values were 17.10±6.2 (11.5-25.2) mmHg with the Icare Pro and 14.01±3.4 (7-24) mmHg with the Icare One tonometer. Comparison of the two methods with Levene’s t-test revealed that the mean difference between the values was -3.08±0.6 with a 95% confidence interval of (-4.39 -1.78), which was a statistically significant difference (p<0.001). Mean IOP measured with the Icare One tonometer was found to be about 3 mmHg lower than the mean IOP measured with the Icare Pro tonometer.

## Discussion

Accurate measurement and regular monitoring of IOP are important in the diagnosis, follow-up, and treatment of glaucoma. The GAT, developed by Goldmann and Schmidt, is widely accepted and is still used as the gold standard method for the measurement of IOP.^1,2^ The Icare tonometer is a small, portable, easy-to-use device that enables measurement without the use of biomicroscope or anesthetic, provides rapid results in uncooperative patients, and is useful in daily routine clinical practice. It is especially convenient for children, individuals with deep-set eyes, and patients who have poor mobility or cannot be examined at the slit-lamp due to physical problems.^5,6^

Studies in the literature comparing the Icare Pro tonometer and GAT reported that IOP measurements obtained with the Icare Pro tonometer were 0.1-3.36 mmHg higher.^7,8^ Vandewalle et al.^9^ and Munkwitz et al.^10^ reported a high correlation and no statistically significant difference between mean IOPs with the Icare tonometer and GAT in glaucoma patients. Pakrou et al.^11^ also reported that mean IOP was measured as 18.2 mmHg by GAT and 17.6 mmHg with the Icare tonometer, with high correlation (r=0.95).Brusini et al.^2^ compared Icare Pro and GAT measurements in a study of 178 primary open-angle glaucoma patients and reported that there was a statistically significant correlation between the measured values, but noted that measurements were affected by central corneal thickness.

The Icare One tonometer is a home tonometer designed for patients to measure their own IOP. Self-tonometry is important for enabling the evaluation of the IOP-lowering effect of treatment and demonstrating diurnal IOP fluctuations. Moreno-Montañés et al.^4^ compared GAT, Icare Pro, and Icare One tonometer measurements in 60 healthy individuals and 90 glaucoma patients and reported no significant difference between GAT and the Icare Pro tonometer, while IOP measurements obtained with the Icare One were an average of 0.3 mmHg higher compared to the other two methods.

Witte et al.^12^ compared Icare One and GAT measurements in 40 glaucoma patients and found that they were significantly correlated in the <60 age group, but not in the >60 age group. They reported that adults over the age of 60 may have difficulty using the device properly, and that tremors and other systemic conditions seen in older adults may reduce the utility and reliability of the device in these patients. In another study comparing Icare One tonometer and GAT measurements, Rosentreter et al.^3^ evaluated 74 glaucomatous and 52 nonglaucomatous right eyes of 126 patients. Among the 95 patients (75.3%) that were able to use the Icare One tonometer and were included in the study, the mean IOP difference between the Icare One and GAT was 0.6 mmHg. In addition, a survey about the use of the Icare One tonometer conducted among the study participants revealed patients aged 70 years and older considered the device difficult to use. Halkiadakis et al.^13^ reported that the mean IOP measured by Icare One tonometer was 2.3 mmHg higher than those obtained with GAT in 60 healthy individuals. Gandhi et al.^14^ compared IOP measurements made with Icare One tonometer and GAT in 60 children with diagnosed or suspected glaucoma. Icare One tonometer measurements were taken twice, once by a clinician and once by a family member. Clinician-measured Icare One IOP values were 3.3 mmHg higher on average than GAT measurements. Measurements obtained by the clinician with the Icare One tonometer were an average of 1.9 mmHg higher than those obtained by the patient’s family. In addition, families were surveyed in the study about the use of the Icare One tonometer and 98% of the participants stated that the device was easy to use.

In the present study, mean IOP was 17.10±6.2 mmHg with the Icare Pro tonometer and 14.01±3.4 mmHg with the Icare One tonometer, with a statistically significant mean difference of -3.08±0.6 mmHg (95% confidence interval: -4.39 – -1.78; p<0.001). The discrepant IOP values obtained with the Icare One tonometer likely stem from inability to use the device properly or obtain measurements from the correct location, or may be related to using the device for the first time. In order to accurately measure IOP with the Icare One tonometer, it must be held horizontally during measurement, but the device does not have any indicator of its position. To avoid the effect of device orientation on IOP values, an updated version of the Icare One, called the Icare Home, equipped with an eye recognition system and position sensors, has recently been introduced to the market. Due to its position sensors, the Icare Home will not obtain measurements if it is not in a horizontal position. In addition, the Icare Home gives an error signal and does not take measurements if the probe is too close to the eye or if the patient’s hand or hair comes between their eye and the probe. In addition, while the Icare One shows IOP measurements as being in a certain range by classifying the values into 11 categories, the Icare Home does not have a display that shows the measured value. Icare Home measurements can be viewed by connecting the device to a computer with the appropriate software.^15^ In our study, we cannot be sure that the participants using the Icare One were holding the device in the proper horizontal position, due to its lack of position sensors. In addition, reduced IOP due to the accommodative reflex may have also contributed to this difference.^16,17^

## Conclusion

IOP measurements obtained at home using the Icare One tonometer can provide guidance in the follow-up of glaucoma patients. However, the reliability of the Icare One is reduced by older adults’ difficulties using the device and the inability to prevent position errors by the user. Because the sample population in this study was small and included younger adults, our findings should be supported by larger and more inclusive patient series.
